# Everyday Nationalism in Unsettled Times: In Search of Normality during Pandemic

**DOI:** 10.1017/nps.2020.40

**Published:** 2020-05-22

**Authors:** J. Paul Goode, David R. Stroup, Elizaveta Gaufman

**Affiliations:** 1 University of Bath, Bath, UK; 2 University of Manchester, Manchester, UK; 3 University of Groningen, Groningen, NL

**Keywords:** everyday nationalism, practice, performance, consumption, everyday foreign policy, COVID-19

## Abstract

Pandemics and other crisis situations result in unsettled times, or ontologically insecure moments when social and political institutions are in flux. During such crises, the ordinary and unnoticed routines that structure everyday life are thrust into the spotlight as people struggle to maintain or recreate a sense of normalcy. Drawing on a range of cases including China, Russia, the UK, and USA, we examine three categories of everyday practice during the COVID-19 pandemic that respond to disruptions in daily routines and seek a return to national normality: performing national solidarities and exclusions by wearing face masks; consuming the nation in the form of panic buying and conspiracy theories; and enforcing foreign policies through social media and embodiment. This analysis thus breaks with existing works on everyday nationalism and banal nationalism that typically focus on pervasively unnoticed forms of nationalism during settled times, and it challenges approaches to contentious politics that predict protest mobilization for change rather than restoration of the status quo ante. In highlighting the ways that unsettled times disrupt domestic and international structures, this work also presents a first attempt to link everyday nationalism with growing work on international practices.

## Introduction

During the long pandemic season of 2020, the most common question has been, “When will things get back to normal?” Arguably, the next most common question has been, “*How* do things get back to normal?” followed closely by, “Will things *ever* get back to normal?” and, finally, “Do we *really* want to go back to normal?” Answering any of these questions is not just a function of epidemiology, as they involve an implicit suggestion that there is an agreed-upon sense of what constitutes normality. People frequently express their sense of a lost normality in terms of the everyday practices and routines associated with daily national existence, such as working, shopping, voting, studying, or socializing. The reality of a life constrained by lockdowns, quarantine, and self-isolation leaves people trapped in worlds which are familiar yet just different enough that they cling to the select few routines they consider essential to their identities—not unlike the ways that migrants cling to the routines they associate with their national identities. The pandemic thus transforms the background practices and routines of everyday life, suddenly conferring new significance on doing normal things as a way of performing one’s nationhood and calibrating national solidarities.

The spatially and socially confining response to the COVID-19 pandemic is qualitatively different from other kinds of disruptive events or disasters that induce protest by disrupting people’s everyday routines (Snow et al. 1998). For some time, scholars working on banal nationalism and everyday nationalism have argued that there is a continuous relationship between everyday nationalism and mobilized nationalism—that is, between the ways that nationalism is activated as a mobilizational frame during contentious political cycles and, vice versa, when nationalism is demobilized and fades into a background as a form of legitimation for new regimes (Goode 2020a; Jones and Merriman 2009; Skey 2009).^1^ Owing to the imposition of lockdowns and quarantines around the world, however, the process of framing nationalist responses to the pandemic are frozen at the point that people start acting consciously in the name of the nation but stop short of mass mobilization.

The COVID-19 pandemic is thus a uniquely disruptive kind of event that brings everyday nationalism to the fore. This article first examines the nature of everyday nationalism as a form of social practice that is commonly observed during settled times and considers how it transforms (and is transformative) during unsettled times like the ongoing pandemic. We next consider specific categories of practice, looking at ways that performance and consumption aim to recover normality. Finally, we build bridges with the international relations literature on international practices by looking at the intersection of everyday nationalism with foreign policy. The article concludes with a brief discussion of the implications for understanding the relationship between everyday nationalism and social movements for future global challenges to normality, like climate change, and for the development of everyday nationalism as a scholarly approach.

## Everyday Practices, Unsettled Times, and the Pursuit of Normality

Everyday nationalism is an approach to observing how ethnicity and nationhood are manipulated as categories of social practice and reproduced “by ordinary people doing ordinary things” (Fox and Ginderachter 2018, 547). It follows in the vein of constructivist works that disaggregate (rather than operationalizing and reifying) social identities, taking up Rogers Brubakers’ (2004) recommendation that scholars focus on *groupness* rather than groups. The crucial methodological move made by this approach is “to replace individuals or groups with ethnic or nationalist practices as units of analysis” (Goode and Stroup 2015, 8). A social practice is “a routinized type of behavior which consists of several elements, interconnected to one other: forms of bodily activities, forms of mental activities, ‘things’ and their use, a background knowledge in the form of understanding, know-how, states of emotion and motivational knowledge” (Reckwitz 2002, 249). Practices relate to specific forms of knowledge that render them recognizable, demarcating communities by their performance and enabling judgments of agents’ competence to perform them.

Much of the literature on everyday nationalism focuses on exposing the ways that people infuse everyday routines with ethnic or nationalist content. The nation is not something that objectively exists but rather functions as a “cognitive frame through which people apprehend social reality and construct routinized strategies of action” (Bonikowski 2016, 429). As Adler and Pouliot observe, “The performance of practices in socially recognizable ways is the source of ontological stability in social life. At the same time, however, it is also from practices that social change originates” (2011, 18). While Jennifer Mitzen (2006) and other proponents of ontological security as a concept (Kinnvall 2004; Rumelili 2015; Subotić 2016) advocate moving from an individual to a state level of analysis, we argue that ontological security in unsettled times offers explanatory power at the level of individual practices, where it originates. Ontological security is inextricably linked to routine practices (Skey 2010; Subotić 2016), whether domestic or international. As Mitzen and Larson (2017) note, most people are ontologically secure and reify their identities passively through daily routines. However, unsettled times bring out the need to reestablish structural relations that have dislocated practices.

There is a clear temporal dimension to practice insofar as it engages with the material world and contributes to social structuring. If this temporal dimension is mostly unnoticed in ordinary times, it is thrown into sharp relief when the structures that contextualize everyday practices come unglued. The temporal dimension of practice explains the disruptive nature of unsettled times. In his review article on nationalism in settled times, Bonikowski describes settled times as “periods when disruptions of varying magnitude […] are absorbed by existing institutions instead of generating widespread social and political transformations” (2016, 427). This perspective is helpful in suggesting that settled times are structured by resilient social and political institutions, though it also assumes that we already know whether institutions will survive.

In contrast to settled times, unsettled times are ontologically insecure moments when everything appears to be in flux. They are defined by the experience or perception of uncertainty regarding the future of social and political structures within which routine social practice takes place and derives meaning. War and revolution are extreme examples, but one might also include global pandemic as such a moment, when the structure of domestic and international politics becomes radically uncertain. The imposition of social distancing, lockdowns, and quarantines not only alter social and economic behaviors but call into question the ongoing viability of both domestic and international institutions over the management of the outbreak. During the COVID-19 crisis, a multinational study recently released by Romir Holding and Boston Consulting Group (BCG) found that upwards of 75% of respondents believed the world was in grave danger and reported that their daily lives had changed as a result of the coronavirus (see Table 1). In the absence of daily work, school, shopping, sports, and other routines, it became common for people to report on social media the sensation of timelessness or loss of normal time. One historian even joked that we should soon expect “Eric Hobsbawm’s new volume, ‘The Age of Eternity: March 2020–March 2020.’ ”^2^

**Table 1.** Perceptions of Threat and Change as a Consequence of the Coronavirus. (“COVID-19 – Srez potrebitel’skikh nastroenii No. 1: Rossiiskie potrebiteli – novaia real’nost’,” ROMIR, April 2020. http://romir.ru/download/BCG_Romir_Covid.pdf.)

	Russia	China	India	Brazil	Philippines	Malaysia	USA	Italy
The world is in grave danger because of the coronavirus	76%	88%	83%	86%	86%	86%	79%	86%
My daily life has changed because of the coronavirus	75%	81%	85%	93%	85%	85%	84%	91%

In practical terms, everyday nationalism in unsettled times manifests in attempts to restore normality, as people seek to recreate everyday routines outside of the social structures that are associated with (and reproduced by) them. In this sense, the perceived relationship between structure and routine in the process of meaning making is inverted in unsettled times: rather than interpreting the meaning of social practices in relation to social structure, social practices seek to affix national meanings to social structures that are in flux. In other words, everyday nationalist practices aim to recreate, reproduce, preserve, or maintain those routines associated with the nation’s normal existence. It is in such moments that the agency of everyday nationalism takes center stage in the performance of national routines. When forced to make hard choices about which routines to preserve, their choices reveal the value placed on performing or consuming national routines. If nationalism is the idea that national membership matters most among all other identities “when the chips are down,” then it is also to be found, say, in the ways that the English panic buy frozen chips, that Russians panic buy buckwheat, or that Americans panic buy handguns.

The following sections unpack examples of how everyday nationalist practices seek to restore national normality and national solidarities. These examples are not meant as systematic research but rather as attempts to probe the value of observing such practices and to suggest an agenda for future research on everyday nationalism in unsettled times. We begin with a focus on two commonly observed forms of everyday nationalist practices: performance and consumption.

Performing the nation relates to ritualized performances in everyday life. National symbols are pervasive in daily life, while rituals provide the connective tissue that link together symbols with national attachments. States regularly organize ritual observances that are meant to naturalize and reinforce the bond between state and nation. However, most people do not experience them as the collective effervescence described by Durkheim, instead injecting their own—often unintended—meanings into these performances of the nation. A rare exception are mass sporting events like the Olympics or the World Cup, which structure the experience of sporting competition around national allegiances (Fox and Miller-Idriss 2008, 547–548). In this sense, it is symbolically and practically significant that the COVID-19 pandemic forced the postponement of the Tokyo Olympics until 2021. In the absence of such visible events at which all members of the nation are at least imagined as figuratively present, performing the nation takes on a different dimension—for instance, in the hand wringing over the visible absence of co-nationals, in demands for their return, in the search for ways to replace the simultaneous experience of nationhood, and in the exclusion of those who are not perceived to belong.

Consuming the nation relates less to the collective sensation of nationhood achieved through performance than to consumption as a means to realizing “the quotidian experience of sameness” (Fox and Miller-Idriss 2008, 550). Consumption involves a range of behaviors through which the nation is commodified and national solidarities are defined. National attachments are expressed through consumption of vital products, which seemingly nobody can do without (as with the aforementioned example of frozen chips in England), through the kinds of media that are routinely read or viewed, or through leisure activities like tourism or gaming. The quintessential form of consumption in unsettled times is panic buying, though increasingly widespread is the consumption of disinformation and conspiracy theories that have accompanied the related rise of exclusionary nationalism with populist and authoritarian regimes (Bieber 2020).

At its core, everyday nationalism dovetails more broadly with practice theory and therefore shares much in common with the practice turn in international relations, which concentrates on the socially meaningful patterns of action at the heart of international politics (Adler-Nissen 2016; Adler-Nissen and Pouliot 2014; Bueger and Gadinger 2018). Though these literatures have developed largely in isolation, this core theoretical relationship becomes particularly visible during unsettled times: though everyday nationalism as an approach has focused predominantly on the national, unsettled times brought on by phenomena like the COVID-19 pandemic or the impending climate crisis (Conversi 2020) blur the distinction between national and international, especially in the ways that transnational solidarities and tropes link domestic and international politics (Goode and Stroup 2015, 17).

## Performing and National Belonging: Pride, Protest, and Face masks

**Figure 1.**
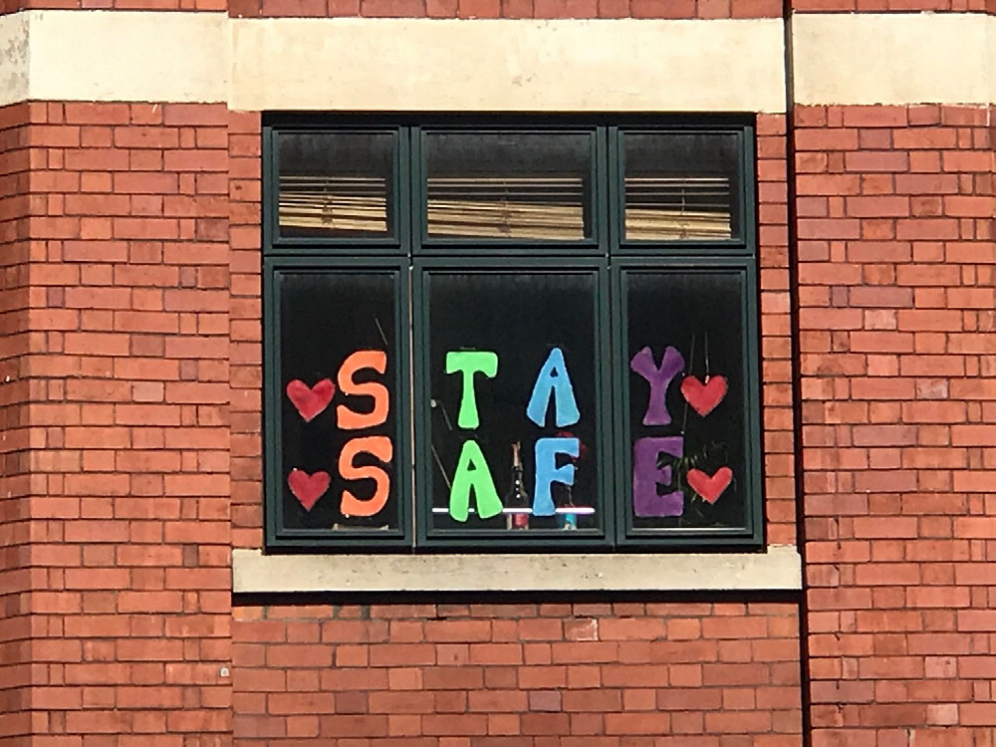
Messages of support for the NHS seen in apartment windows in Manchester’s Northern Quarter on April 8, 2020.

At 8 in the evening of March 26, 2020, people throughout the UK threw open doors and windows, and stepped out onto balconies to applaud. The gesture of gratitude, given under a stay-at-home order issued earlier in the week and occurring as death tolls from the virus in the country hit 100, sought to thank the national heroes of the National Health Service (NHS) working to combat COVID-19. Up and down the country, Britons made noise in support. In Scotland, bagpipers played tunes loudly in appreciation. In Manchester, residents banged on pots and pans. Key monuments—the London Eye, the Tower Bridge, and the Shard in London—all lit up blue in solidarity with carers. In windows across the country schoolchildren placed drawings of rainbows, with messages of love and support (see figure 1). The tremendous show of gratitude rapidly took on a larger meaning as a symbolic show of resolve, unity, and sacrifice of the British people (Mohdin 2020; *BBC News* 2020a).

Photo by David Stroup.

Such effusive performance of thanks and unity stood as expressions of national character in the face of desperate challenges. Indeed, performances of collective effervescence like the “Clap for Carers” events intend to produce feelings of national solidarity and common belonging that provide ontological security, especially in times of hardship or imminent crisis where instability and insecurity prevail (Durkheim 1915; Goode 2016, 435–437; Skey 2011, 95–118). Likewise, these performances also became a way of justifying (or perhaps even rejecting) the changes wrought by the virus on the course of everyday life. By standing up to applaud for care workers, participants sought to recognize, validate, and participate in the efforts to beat the virus and restore normality.

Simultaneous to these outpourings of national gratitude and solidarity, another, more contentious form of national identity performance unfolded. In cities in over a dozen states across the USA, protests cropped up outside state houses and governors’ mansions demanding an end to stay-at-home orders and insisting that businesses be reopened (Rose 2020). As some protestors circled the buildings in their cars, honking their horns and shouting slogans out of open windows, others lined the sidewalks carrying handmade signs and American flags. Others, taking matters further, carried weapons. In Austin, Texas, famed right-wing media personality Alex Jones led protestors in a chant of “Let us work!” Watching from the White House, President Donald Trump expressed sympathy, claiming of the protestors, “Their life was taken away from them” (*BBC News* 2020b). In Jefferson City, Missouri, one man attending the protests bluntly told a BBC news crew, “We need to reopen the country. We need our lives back. Now” (Maqbool 2020).

In settled times, routine performances of national commemoration become episodic, often unobserved (Billig 1995). However, even if they pass without fanfare or do not move participants, these performances of ecstatic nationalism highlight the basis for national solidarity and communal belonging (Skey 2011, 99). As they become rote, such rituals become “unthinking and unquestioned performance[s] of the nation” (Fox and Miller-Idriss 2008, 549). Crisis events leave a wake of ontological instability by overturning those taken for granted practices of nationhood and challenging the symbolic order established as normal (Skey 2011, 115). Instability foregrounds the kinds of solidarities that usually only achieve low levels of salience. For example, in ordinary times, displaying a flag outside one’s home may be a passive, pervasive, and unobserved invocation of the nation, while during times of crisis flying a flag may become a purposeful, demonstrative act (Skitka 2005). In the atmosphere of uncertainty created by the COVID-19 pandemic, the outpouring of support for essential workers through the performance of communal applause illustrates how ordinary gestures provide a platform for messages of unity and national strength.

However, times of crisis may also invite less inclusive calls to solidarity. When unsettled times place the structures that provide ontological security—especially to historically dominant or majority groups—into flux, everyday practices of identity become radical attempts to reassert a vision of normality. As national identity offers a point of stability in a changing world, such performances often seek to reestablish the symbolic power of the dominant group (Kong and Yeoh 1997, 214; Skey 2011, 108–117). Protestors’ framing of the lockdowns as a betrayal of the American way of life, and parading with flags and signs that invoke national symbols in support of their demands to reopen the country, illustrates one such attempt to reestablish normality by invoking the nation in performative terms. Usually such performances call for national solidarity to reclaim stability, while also excluding those outsiders who are seen as perpetuating instability (Skitka 2005).

### Wearing Face masks and National Belonging

During the COVID-19 pandemic, the outbreak of harassment of people of Asian descent living in western countries—particularly in the UK and USA— clearly illustrates such radical assertions of solidarity and exclusion (Tavernise and Oppel 2020). Many victims felt their decisions to wear masks in public signaled them out as targets for abuse. Why did mask wearing become a signifier of national identity? Daily acts of performance related to attire provide many of the most visible and readily apparent signifiers of national belonging and display “social information relevant to the public interest” (Maxwell 2014, 7, 46–58). In unsettled times activists may weaponize such sartorial features, turning them into a means by which to exclude others or casting them as suspect or dangerous (Haddad 2007; Gohil and Sidhu 2007). In the unsettled context of the pandemic, the sight of people wearing masks in public stood out as a sign of abnormality. Dominant majority groups in these countries, driven by bigotry and viewing the virus as brought in by foreigners from Asia, blamed those Asians wearing masks for spreading the virus.

In East Asia, mask wearing stands as a feature of daily life that predates the current crisis. Historical records suggest that frequent mask-wearing became a widespread practice in China after the 1910 Manchurian plague epidemic, and in Japan by 1918 following the Spanish flu pandemic (Burgess and Horii 2012; Lynteris 2018). More recent outbreaks of SARS, H1N1, and MERS reinforced norms about mask wearing in the region (Syed et al. 2003; Tang and Wong 2004; Siu 2016).

Face masks achieved the “transformation of their wearers into ‘reasoned’ subjects of hygienic modernity” (Lynteris 2018, 443). Masks transformed not just individual wearers but also societies on the whole. Looking back at the 1910 plague, Lynteris remarks that in popular rhetoric, the antiplague masks provided “indisputable, photogenic proof of Chinese scientific sovereignty” (452). Examining the responses of Hong Kongers to the 2003 SARS epidemic, Siu found a similar connection between mask wearing and civic duty. A respondent plainly summarized this sentiment, claiming:

Wearing a face mask was a way to tell others that you were exercising civic responsibility and that you cared a lot about Hong Kong. You did not want to spread the virus to others, so you wore a face mask. You wanted to tell others that you were on the same team with other Hongkongers in fighting against SARS, so you wore a face mask. Wearing a face mask was a sign of solidarity and civic responsibility at that time.

(Siu 2016, 7)

Following the SARS outbreak of 2003, mask wearing became a common practice in much of East and Southeast Asia even outside of health crises. In China, surgical masks became a means of dealing with smog and air pollution in urban environments, while in Japan the practice of wearing courtesy masks to protect others from illness became so common that surgical masks came to be considered a fashion accessary (Eveleth 2019; Gordenker 2014). During the COVID-19 pandemic, the daily habit of mask wearing became an extraordinary gesture in the effort to beat the disease. By simply wearing a face mask, ordinary citizens could contribute to ending the pandemic and regaining stability. Mask wearing became an integral part of public health campaigns to combat the virus in much of East and Southeast Asia.

**Figure 2.**
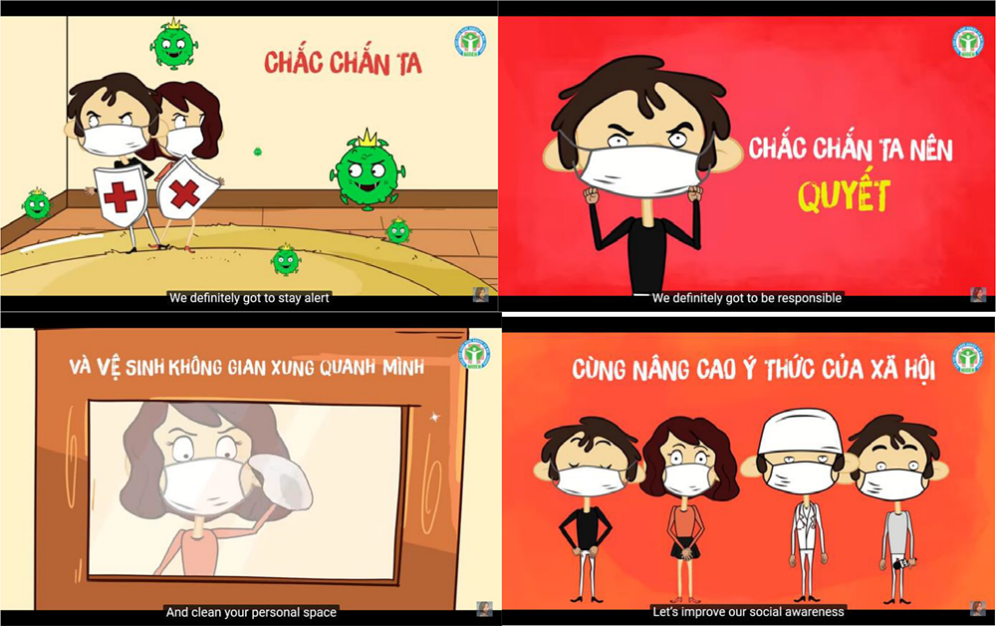
Screenshots from the Vietnamese music video for “Jealous Coronavirus” (“Ghen Cô Vy 2020”) with English subtitles.

For example, in Vietnam, the Ministry of Health produced a public service announcement about best practices for keeping safe in the form of a three-minute pop song called “Jealous Coronavirus” (“*Ghen Cô Vy*”) sung by pop stars Khắc Hưng, Min, and Erik, and set to an animated video. The lyrics implore viewers to “push back the virus” by taking various precautions, including handwashing and “clean[ing] your personal space” (figure 2). The cartoon characters in the video are shown scrubbing down floors and windows, all while wearing face masks. The song fueled a viral dance meme—often featuring masked performers—whose moves illustrated how to properly wash hands and observe social distancing (Benner 2020).

In places like Vietnam, Japan, South Korea, Hong Kong, and China, where mask wearing became a critical part of virus response, the act became a performance of national solidarity. As Feng et al. suggest, adopting a policy that promotes universal face mask wearing “prevents discrimination of individuals who wear masks when unwell because everybody is wearing a mask” (Feng et al. 2020). A face mask became part of the uniform of a patriot. By wearing a mask in public, citizens performed national unity and demonstrated their willingness to work together as the community of the nation to prevent the spread of the virus and reestablish the conditions of normal life.

### Wearing Face Masks and National Exclusion

Elsewhere, mask wearing provoked a different kind of performative nationalism. In countries outside of Asia, as governments stressed that citizens should refrain from hoarding masks in order to preserve a stockpile for medical professionals, masks were regarded as being worn by those already carrying the virus (Feng et al. 2020). Wearing a mask in a public place became a symbol of the abnormal circumstances created by the pandemic, reproducing anxieties about ontological and physical security. As media outlets reported that COVID-19 originated in China, Asian people in other countries began to report being scapegoated for spreading the virus and receiving abuse for appearing Chinese. In this context, the practice of wearing a mask also exposed Asian wearers to harassment by dominant groups accusing them of causing the pandemic. Reporting on the outbreak of discriminatory acts triggered by the virus in the UK, *Guardian* dubbed the rising tide of incidents of anti-Asian racism—particularly those which targeted Chinese students—“maskaphobia” (Weale 2020). Accounts of harassment filed to the Stop AAPI Hate reporting system, a joint project of the US-based Asian Pacific Policy and Planning Council (A3PCON) and the Chinese for Affirmative Action (CAA), illustrate how such performances of exclusion made Asians the target of harassment as the COVID-19 pandemic spread. In the two weeks between March 19 and April 1 alone, eyewitnesses in the USA filed claims for 1,135 incidents of harassment against Asians (Jeung 2020a; Jeung 2020b).

The reports suggest that in 14% of incidents harassers singled out mask-wearing Asians in public places (Jeung 2020a). One victim described being intimidated by a driver of another car who tried to cut them off in traffic because they wore a mask while driving. Another reporter who wore a mask while riding the train system in the Bay Area of California described being verbally assaulted by five teenagers who then hit the passenger with their backpacks. A respondent who wore a mask while working at a yoga studio in Irvine, California, described being verbally assaulted by two white patrons who claimed, “Chinese people have ruined Irvine [by being] so dirty.” One of the white women said whites had made a mistake by letting Asian “garbage” into Orange County (Jeung 2020b). Conversely, those not wearing masks also found themselves victims of harassment. One victim of verbal assault reported, “As I was walking to my bus, a white, middle-aged man screamed at me to ‘wear a respirator’ because I’m Asian” (Jeung 2020a). Another woman recounted an incident in which two white women pantomimed covering their faces as she entered the train:

I walked into the train carriage and immediately two teenage girls started screaming and eewwwwing and making a show of covering their mouths and faces with a scarf then stood up and ran to the other end of the carriage (which was more crowded) jeering at me.

(Jeung 2020b)

In both cases, masks were signaled out by harassers as markers of difference and implied foreignness. To those committing harassment, wearing a face mask became so evidently a marker of foreignness that even the absence of a mask becomes an act of national significance, implying that those Asians not wearing one might be actively spreading the virus.

Frequently, such incidences of harassment became acts of performance in and of themselves. Several victims reported that harassers performed the act of being sick on them, often by coughing at them. Such incidences were often followed by laughter or further attempts to intimidate the victim, such as glaring at them:

While we were passing a group of four men, one of them coughed into me, not once, but TWICE, without covering his mouth. As I turned my head back, they all burst out laughing.

(Jeung 2020a)

The performance of coughing or spitting on Asians, particularly those wearing masks, conveys a forceful and exclusory message that those targeted do not belong to the community and are thus not afforded solidarity. Such gestures imply that those targeted are carriers of the disease who brought it into the community, painting them as responsible for the upheaval that overturns the ability to live a normal life. Though actions such as wearing a face mask may be utterly banal in ordinary moments, during the COVID-19 pandemic such practices became transformed into deeply significant markers of estrangement from the community of the nation. By wearing masks in public these people became a manifestation of the ontological insecurity felt by harassers. In performing acts of harassment to exclude Asians, harassers attempted to reassert power hierarchies, restore what they considered to be normal visions of the nation, and forcefully exclude those they blamed for disrupting the practice of everyday life.

While many of these incidences of harassment merely implied this exclusory understanding of national solidarity rather than stating it explicitly, some made direct appeals to national self-defense. As an example, on March 30, 2020, an unidentified, white Australian man brandishing a bullwhip stalked back and forth on the sidewalk outside the Chinese consulate in Sydney, shouting racist epithets about Chinese people and menacing Asian-looking passersby. Eyewitness reports emphasized that the man wore an Akubra, a wide-brimmed, rabbit fur, felt hat considered by many as a symbol of rural working-class Australians. Cracking the whip and shouting in the direction of Chinese nationals queued up waiting to enter the consulate and those walking by wearing face masks, the man accused them of having “spread that fucking filthy disease worldwide” and “trying to take over the world.” He also made pleas to Australians, shouting, “No more shit from China! Wake up Australia! Be wise to these pricks!” (McPhee 2020). By wearing a hat symbolic of Australian national identity and making direct appeals to the Australian people, the man’s performance not only embodies a vision of how Australians should dress and act but also weaponizes marks of otherness—such as wearing a mask—against the Other he claimed sought to subjugate, or infect, Australians. While the man’s actions may stand outside of everyday practice, they reflect the ways in which the virus heightens concerns by confirming prejudices and convictions that the status quo ante was already flawed or endangered. The protestor’s vision of returning to normality begins with a radical change of immigration and economic policy.

Instances like the harassment in Sydney reveal much about the way in which everyday actions become conflated with nationalist tropes about unity, sovereignty, and threat. The outbreak of COVID-19 came amidst increasing tensions between China and Western liberal democracies. In the USA, the long-running conflict with China over trade heightened accusations and blame between both states. Donald Trump’s continual labeling of COVID-19 as the “Chinese virus” no doubt raised tensions and inspired imitations of such rhetoric among ordinary people in their daily lives (Rogers, Jakes, and Swanson 2020). In Australia, the virus spread amidst concerns about China’s influence in Australian domestic matters (Brophy 2018; Hamilton 2018). In both cases, the presence of Chinese people—or even those that appeared Chinese—threatened understandings of the majority that formed the basis for ontological security. In calling explicitly for national solidarity on terms that excluded those who could be scapegoated as foreign transgressors, harassers asserted a radical understanding of normality which deliberately excluded those whose presence they claimed overturned the habits of daily life and upset the foundations of the nation itself.

## Consuming: Panic Buying, Conspiracy Theories, and National Anxieties

In the early hours of a Monday in mid-February, three men executed a daring robbery in the Mong Kok district of Hong Kong, which has a history of triad gang activity. Armed with knives, they threatened a delivery man and escaped with 600 rolls of toilet paper, worth approximately US$130. Police found the stolen toilet paper later the same day in a nearby guesthouse, arresting two members of the gang while a manhunt continued for the third (Ho-him 2020). The robbery came two weeks after the circulation on messaging apps of a fake memo stating that toilet paper production had been halted owing to factory closures in mainland China. Panic buying ensued, and then it spread. In Australia, panic buying of toilet paper brought out the police to settle disputes in the aisles of grocery stores, a delivery truck catching fire made headlines, and a provincial newspaper published eight blank pages “to give the nation what it wanted” (Kaye and Gallagher 2020).

Tales of panic buying, and even armed theft of toilet paper, were met with bemusement elsewhere in the world and then with resignation as Europeans and Americans started hoarding—after all, perhaps the most essential of daily routines was at stake. Yet rather than encourage a sense of universal togetherness, the experience of panic buying draws stark boundaries around national solidarities while highlighting patterns of national exclusion.

### Consumption and Everyday Nationalism

Consumption is laden with power relations that define, reinforce, and communicate social identities. Access to goods and services varies not just with income but with class, race, ethnicity, gender, and citizenship. In a globalizing world, the interaction of consumption with intersectionality is not coincidental. Rather, consumption is bound up with the mass experience of marginality, “a cultural activity of the non-producers of culture […] who nevertheless buy and pay for the showy products through which a productivist economy articulates itself” (de Certeau 1984, xvii). This mass experience of marginality was vividly displayed with the collapse of communism, as millions of citizens in former socialist states grappled with new consumer practices imported from the West (Berdahl 1999; Humphrey 2002). Consumption further reifies and changes social identities, such as the way that ethnotourism provides a lifeline to struggling communities at the same time that it wrests control over ethnic authenticity from its putative owners (Comaroff and Comaroff 2009).

Yet even the powerful (elites or social majorities) struggle with the commodification of national identities, which invariably entails the domestication of global brands and their competition with local goods and services (Edensor 2002, 112). In settled times, the marketing of goods and services often draws upon consumer nationalism and national stereotyping by advertising countries of origin. Sometimes this is explicitly marketed, as with “Made in the USA” stickers and signs. Russia’s adoption of import substitution in response to Western sanctions (and Russia’s own countersanctions) similarly was packaged as patriotic support for domestic producers (Balmforth 2015). Some products are promoted in ways that associate goods with national brands (German cars and cutlery, Swiss watches) or with national imaginaries (rolling hills and pastoral images for agricultural and dairy products). In what DeSoucey (2010) depicts as “gastronationalism,” states consciously act to institutionalize their national brands and to protect domestic markets by laying claim to the geographic and cultural production of certain foods.^3^ Similarly, foreign policies can powerfully connect other nations with goods or products through bans, such as Russia’s various bans on Belarussian milk, Georgian wine, Ukrainian chocolate, or Turkish tomatoes.

Crucial to these efforts is the routine nature of consumption. Edensor observes that “shopping for things is most frequently a familiar, mundane activity, necessary for the reproduction of self and household, as well as a means of experiencing pleasure, marking status, and expressing identity” (2002, 110). Changes in shopping routines can symbolically and politically reinforce or contest national identities, particularly when they are challenged by external forces. Caldwell’s (2002, 307–309) examination of food practices in Moscow discovers that shoppers put a premium on domestic foods with a tie to the Soviet past as a way of appealing to national pride while resisting transnational influences. In her examination of Ukrainians’ boycott of Russian goods in 2014 as a form of “patriotic consumption,” Bulakh observes that “commodities are perceived as physical continuations of the state and nation (Ukraine) and/or embody the enemy state (Russia)” (2018, 82).

### Panic Buying and Fantasies of Collapse

Panic buying is a distinctive form of consumption that arises during unsettled times. As a response to the perceived threat to ontological security posed by the pandemic, panic buying reflects people’s desires to ensure or restore normalcy in their daily routines beyond concerns about mere survival. While the dynamics of panic buying might be likened to a bank run on essential goods, such metaphors conceal the significance of consumers’ selectivity of goods or shopping locales. In other words, what counts as essential is not merely an indifferent calculation of nutritional requirements: if panic buying was strictly about serving nutritional needs, then there would not be empty shelves of frozen chips (french fries) in England while frozen Asian vegetables remain in plentiful supply.

In the current pandemic, it has been argued that the designation stocking up might be more accurate than the term panic buying, as individuals initially purchased a small additional quantity of items to see them through a period of self-isolation. However, the cumulative effect of stocking up on just in time supply chains was widespread shortages and hoarding of essential goods (toilet paper, hand sanitizer, rice, pasta, face masks, bottled water, and so forth) in Hong Kong, Taiwan, Singapore, Japan, Thailand, New Zealand, and Australia, before spreading to Europe and the USA. In turn, shortages were attributed to the breakdown of social and political order and informed by collective memories. Russian shoppers rushed to buy buckwheat kasha, still mindful of the rapid inflation of prices in 2014 following the collapse of energy prices and the imposition of international sanctions (Zelenaia 2020), as well as families’ reliance on buckwheat during the hyperinflation of the 1990s. Americans distinguished themselves internationally by rushing to buy firearms, motivated by fears of looting and violence and concerns about the government’s previous inability to manage crises like Hurricane Katrina (Oppel 2020).^4^

Western commentators commonly connected the COVID-19 pandemic with the direst example of social and political collapse in recent memory, likening the shortages and apparently rampant individualism with the collapse of communism. One observer lamented after a trip to the grocery store: “The last time I had seen a food shop with so little actual stuff in it relative to the shelves was on a school trip to Soviet Russia 30 years ago” (Wilson 2020). Similarly, a British analyst even argued, “Thirty years after the demise of their republic, the East Germans can teach us a great deal about how to deal with shortages” (Braw 2020). Another common comparison in the UK was wartime rationing, along with the patriotic subtext that it was Britons’ duty to get by with less in the face of an existential crisis.^5^ For some, panic buying was symptomatic of a deeper crisis of government and concern for survival among those in economically precarious situations, potentially presaging an era of fundamental change or even revolution. Comparing the current crisis to the Great Fear that led to the French Revolution, Croatian philosopher Srećko Horvat argues, “the real fear lies in poverty and unemployment, in the daily unveiling of a system that is broken. And even if we can already see the coming of an even stronger surveillance capitalism, merging of thermal scanners and facial recognition, bio-political control of populations and restrictions on mobility, what if it is precisely this panic and rumor that also contains a potential emancipatory feature?” (2020).

### Hoarders, Hamsters, and Hashtags

Panic buying not only expresses anxieties about the ongoing viability of social and political order but also about the availability of social trust and the willingness to accept ethnic, class, and other minorities as co-nationals. Despite people simultaneously experiencing the pandemic as a national crisis, panic buying is not so much a unifying national experience as it is a means of patrolling the boundaries of national membership and inclusion. Usually it is portrayed as irrational, selfish, and antisocial, with hoarders branded and stigmatized for their national betrayal. In Germany, hoarders were derided as *der Hamsterkauf*, equating panic buyers with nervous hamsters storing food in their cheeks.^6^ As one British columnist lamented, “Panic buying has trampled to death national myths patriots once cherished. We now see that ‘quintessentially English’ does not now mean a reserved character with a stiff upper lip joining an orderly queue. But a demonically possessed shopper lunging towards the last four-pack of loo roll” (Cohen 2020). More devastating was the tearful video posted on Facebook by NHS critical care nurse Dawn Bilbrough, whose condemnation of panic buying of fruit and vegetables went viral (ironically) and forced the UK government to urge shoppers to restrain themselves.^7^ In the US, the story of two brothers who bought 17,700 bottles of hand sanitizer and then sold them online for up to $70 per bottle provoked moral outrage, hate mail, and death threats (Vigdor 2020).

All consumption has a spatial dimension, such as where one chooses to shop, that identifies products and social interaction with normal routines. Panic buying not only disrupts those routines but also highlights hidden, internal forms of exclusion in the linking of the pandemic to an ethnic (usually Asian) Other. One visible manifestation of this linkage is the avoidance of local groceries often run by ethnic minorities in favor of larger (and less ambiguously national) supermarkets. In the USA, the linking of pandemic with ethnicity inflamed anti-Asian sentiments and racism, leaving Asian grocery stores empty of shoppers and relatively unaffected by shortages in major cities like New York and Los Angeles. In Australia, the social media rumor mill alleged that organized busses of Asian or Chinese shoppers were invading small towns and stripping the shelves bare, though journalists could not find any evidence (Meade 2020).

**Figure 3.**
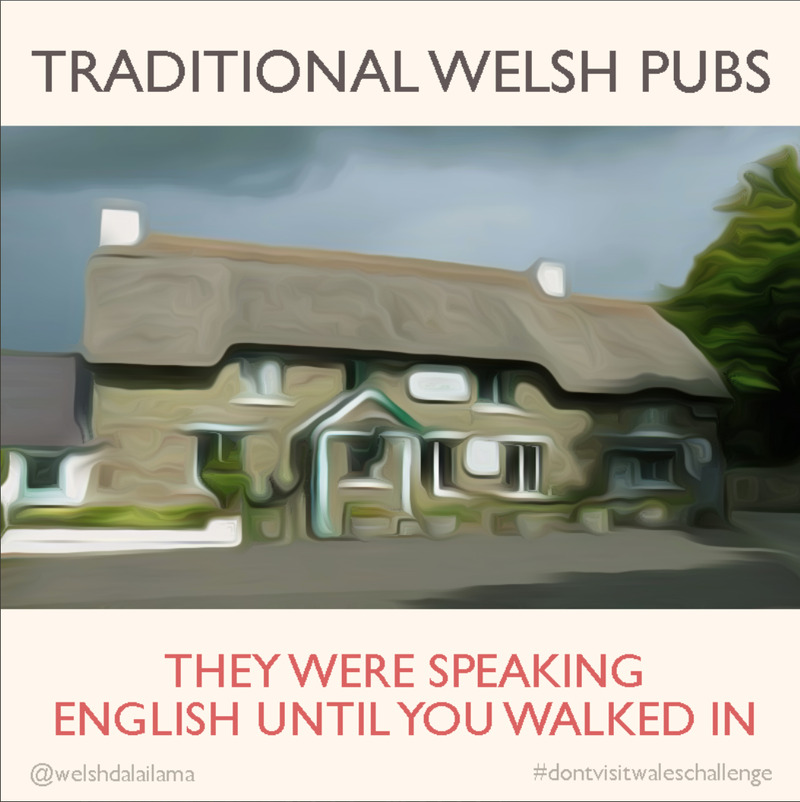
#dontvisitwaleschallenge post on Twitter.

Consumption amid pandemic also foregrounds other forms of internal exclusion rooted in class and urban geography. Large cities and financial hubs were not only the first to be affected, but relatively affluent people sought to escape the outbreak by retreating to beaches and the countryside: Muscovites fled to Sochi and Londoners to Devon, spreading the virus to the provinces and provoking a backlash that fused national identification with class and administrative neglect. In the UK, Scots and Welsh took particular aim at the colonial stereotyping of national landscapes, altering vintage tourism posters to communicate incredulous and even hostile messages on social media to deter would-be visitors from England (see figure 3).

( Original post by Dai Lama [@WelshDaiLama]. “(Don’t) Visit Wales,” Twitter, April 11, 2020, 9:05 a.m., https://twitter.com/WelshDalaiLama/status/1248884952558718976.)

### Consuming Disinformation and Conspiracy

Finally, the lack of information about the virus and the imposition of quarantines around the world have simultaneously increased consumption of online media and provided a convenient platform for spreading and consuming conspiracy theories. Conspiracy theories thrive in environments where there is low trust in government, experts, and traditional media. Various world leaders contributed to the lack of trust by spreading disinformation and opportunistically exploiting the pandemic to enhance their power (Hungary’s Victor Orban), dismissing the severity of the threat (Donald Trump in the USA, Jair Bolsonaro in Brazil, or Boris Johnson in the UK) or blaming it on foreign powers (Iran’s Ali Khamenei). These low trust environments not only encourage conspiracy theories but also facilitate the consumption of fake cures and preventative devices promoted by their purveyors, including Alex Jones’s attempt to sell anti-coronavirus toothpaste (Ferré-Sadurní and McKinley 2020) or Trump’s promotion of hydroxychloroquine.

Russia is a useful (but certainly not the only) case for probing the ways that conspiracy theories are consumed and propagated in low trust environments.^8^ According to a recent Levada Center poll, more than half of Russians distrust official information about the coronavirus (Levada-Tsentr 2020). Russia’s government initially attempted to push ahead with a referendum on constitutional amendments that would ensure Vladimir Putin’s ability to remain in power indefinitely and even encouraged regional governments to link fighting the virus to voting for the amendments, though it was later forced to postpone its plans. It also sought to use the pandemic to speed adoption of digital surveillance technologies, though its ambitions were scaled back after botched implementation (Goode 2020b).

Russian anthropologist Aleksandra Arkhipova characterizes the three phases of conspiracy and myth making in relation to the spread of the pandemic (Nikitinskii 2020). In the first phase (January–February), one finds xenophobic reactions in claims that the virus is spread by bananas or packages from AliExpress. In the second phase (March), hucksters and con artists exploit the public’s lack of trust in government and medical experts, spreading fear and pushing fake cures. Even the Kremlin’s Press Secretary Dmitrii Peskov was discovered wearing a useless “virus blocker,” which he immediately ceased wearing when pressed for details (Kovalev 2020). In the third phase (April), people begin to fear for the future, and conspiracies spread, for example, about the transformation of the country into a digital prison under quarantine. In this fashion, uncertainty about the limits of the government’s ambitions and its push for digital surveillance converge in conspiracy theories about the government’s plan to use vaccinations to secretly implant tracking microchips. In each phase, conspiracy theories and myths circulate in response to the disruption of normal life, provide explanations for how it might return (i.e., by way of fake cures), or propose justifications for taking extraordinary action for fear that things might never be the same again.

## Extending and Embodying Foreign Policy in Everyday Life

“So I called these people warriors. And I’m actually calling now … the nation warriors. We have to be warriors. We can’t keep our country closed down for years. And we have to do something.” Speaking at a face mask plant in Arizona, President Trump portrayed nursing staff as soldiers in the nation’s war on COVID-19 (Oprysko 2020). In turn, invoking the war metaphor seemed to justify reopening the economy: if people die, their loss would be considered acceptable in wartime conditions. Unsettled times—war chief among them—bring about a war rhetoric that juxtaposes enemy and friend, inviting extreme political action that increases the uncertainty. There have been several academic interventions on the harm of war metaphors in the time of the pandemic (Van Rythoven 2020). As Jackson and Laucht (2020) point out, militarized rhetoric helps translate unfamiliar into familiar, thus minimizing the unsettled character of the phenomenon but, at the same time, increases nationalist sentiment associated with the war effort. While the actual enemy is invisible in the context of the pandemic, leaders’ war metaphors demand antagonism to particular groups (foreigners, immigrants, or minorities)—even more so when they insist on calling COVID-19 the “China virus”—as ordinary citizens respond with racially motivated crimes and harassment.

These war metaphors invoke the notion of a nation under siege and suggest that calls on national effort to manage the uncertainty of unsettled times involve combatting a force from beyond the water’s edge. Everyday foreign policy is accorded little attention in the literature on everyday nationalism, though they are closely related. Given that foreign policy emanates from the state, it requires less effort to support than oppose, making it arguably a more inclusive form of civic nationalism (Halikiopoulou, Mock, and Vasilopoulou 2013; Reeskens and Wright 2013). As Prizel notes, “[an] emotional, albeit irrational sense of nation and national identity […] is an extremely important if not driving force behind the formation of […] foreign policy” (1998, 14). Scholars of Chinese nationalism argue that popular nationalism may constrain foreign policymakers, though they focus primarily on instances of mass popular protest rather than emphasize the everyday approach (Gries, Steiger, and Wang 2016). At the same time, observers of China note that hypernationalistic rhetoric is both a top-down and bottom-up phenomenon (Kim 2020), confirming that the population needs to maintain its ontological security in unsettled times. Similarly, the international relations literature discusses foreign policy and performance in terms of diplomatic practices (Urrestarazu 2015; Neumann 2002; Ringmar 2012) or rituals (Hauerwas 2015) with mostly feminist scholarship paying attention to the everyday and grass roots manifestations of international relations (Acuto 2014; Wibben 2016; Björkdahl, Hall, and Svensson 2019).

Everyday foreign policy is tied to everyday nationalism through similar practices involving performance and consumption. After all, both of these are aspects of regular foreign policy where governments offer humanitarian aid or ban imports from certain countries. The literature on (everyday) embodied performance of nationalism (Balogun 2012; Militz and Schurr 2016) is rather limited in comparison to its discursive iteration (Fox and Miller-Idriss 2008; Wodak and Krzyżanowski 2017; Bonikowski 2016). Yet, in unsettled times, physical borders are fortified by physical bodies that can sustain or break with the governmental policy. As noted above, xenophobic street harassment is another (embodied) manifestation of everyday nationalism given that nationalism in general is predicated on designation and exclusion of the Other (Bieber 2020). While citizens often are not privy to government decision making, they are able to practice everyday foreign policy by implementing the government’s decisions on their own accord. People already engage in (and vote for) exclusionary practices, but those practices become significant and meaningful when state policy or capacity are threatened in unsettled times. When things are in flux, ordinary people see their actions as enforcing national policy or defending their ideas about the way the nation ought to be in settled times.

Stigmatizing and discriminating against those perceived as contaminated is also an everyday reflection of state policy, such as in closing borders with certain countries. In this sense, the harassment and racism directed toward Asians in the wake of COVID-19 is continuous with foreign policies that foster internal exclusion. Likewise, the virus exposed older, deeper-seated suspicions of perceived outsiders. One of the historically common forms of exclusion in unsettled times is anti-Semitism (Durkheim [1899] 2008). In times of social change and uncertainty—be it plague, crusades, reformation, modernization, revolution, hyperinflation, or globalization—Jews have always been targeted as the Other. If during settled times Jews were singled out through places where they had to settle, wearing certain clothes, unsettled times brought pogroms, where a perceived alien element had to be forcibly removed from the body politic either by expulsion or by means of physical annihilation. Even in the Middle Ages, the equivalents of doxing and deplatforming preceded physical abuse in the form of pamphlets, carnival plays, and religious literature (Rose 2015). An uptick in anti-Semitism often acts as a canary in the coal mine for other types of (violent) xenophobia that increase in unsettled times. During the COVID-19 outbreak, the Kantor Center at the University of Tel-Aviv reported an 18% uptick in instances of anti-Semitism from the previous year, and the European Jewish Congress noted an increase in incidents in which Jews were blamed for spreading the virus (Heller 2020).

As mentioned above, everyday foreign policy has an important discursive dimension. Here, it is important that a lot of the (nationalistic) identity discourse, even on the grass roots level, relates to foreign policy (Hansen 2016; Morozov 2002). Citizens obviously discuss unsettled times at length online. Broadly, they can be divided into several groups: patriotic groups that look for scapegoats and conspiracy theories to explain why the times are unsettled; conventional nationalists who seek to maintain their ontological routines in the face of crisis; and cosmopolitans that build their ontological security through empathizing with others’ uncertainty and sympathizing with the victims. The first group can probably best be exemplified by the antilockdown protesters in the USA who employ rhetoric from the American Revolution (“Give me liberty or give me death”); blame China (the “Chinese virus”), Bill Gates, or George Soros (the latter being the symbol for anti-Semitic conspiracies around the world) for spreading the virus; and demand that their government “punish China.” Opinion leaders in conservative Russian Orthodox circles have also circulated numerous rumors that the pandemic is orchestrated by the global financial elite (Gaufman and Vovk 2020)—a common anti-Semitic dog whistle—making them ideologically and rhetorically close to US white nationalists.

### Blurring the Domestic and International through Excluded Bodies

Unsettled times underline the links between personal behaviors and international affairs. Feminist scholars have always emphasized that the personal is both political and international (Enloe 1989). This is especially true in the times of a pandemic where education and work spaces have moved into intimate spaces. In this way, the pandemic further confirms the overlapping of the online and everyday spheres, and it suggests they should be studied as such (Franklin 2001). Unsettled times blur the distinction between national and international especially with the domination of online platforms, such as Zoom or Google, that are used across the globe. While most would laugh at the sight of naked husbands or mistresses in the background, or cats and dogs interrupting work calls, the transition to online internationalizes and exposes inequalities as well as the understanding of nation.

**Figure 4.**
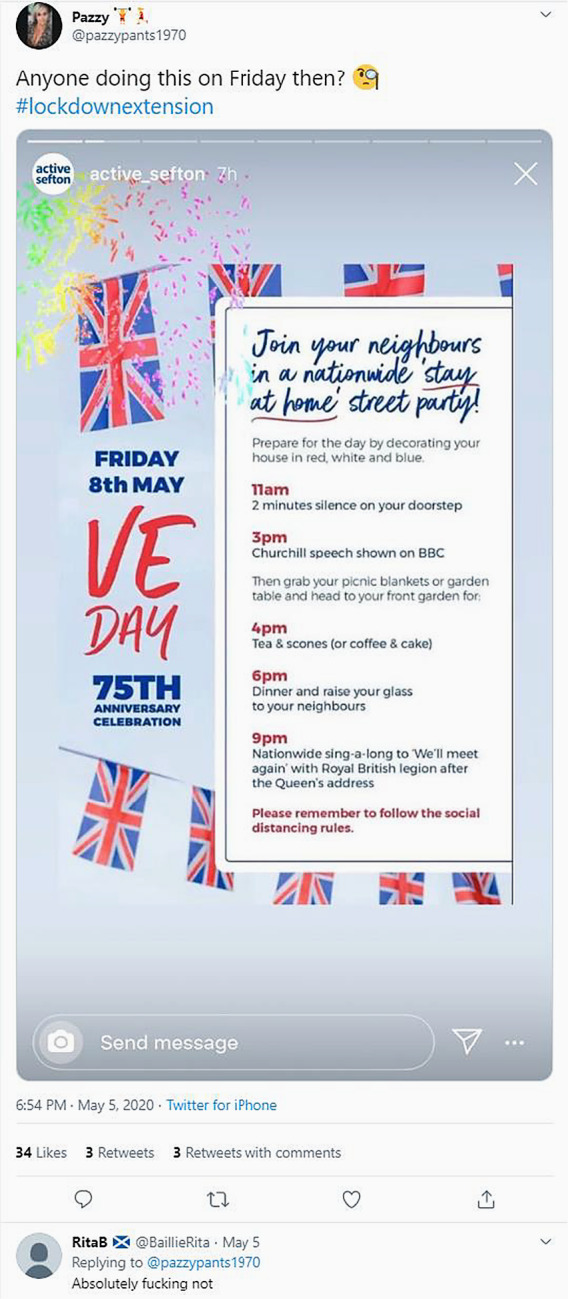
VE Day ‘stay at home’ street party flyer.

With calls to celebrate King’s Day in the Netherlands on the balcony or Victory in Europe (VE) Day in the UK in the back yard, the nation has invaded personal spaces through online communication. For instance, to create an imagined community from a banal nationalist top-down perspective, British councils disseminated posters calling for the VE festivity at home (see figure 4).^9^ To perpetuate a sense of normalcy in observing a national holiday, the stay-at-home party was intended to be a show of national solidarity and inclusion that simultaneously neglected various religions and even classes in the UK. The prescribed practices (taking afternoon tea, listening to the queen, holding a party in the garden, singing pop songs, and decorating the house red, white, and blue) attempted to invoke a safe, secure, comforting vision of Britain. However, the attempt to have a normal observance of the holiday illustrates the ways in which local councils and residents ignore and exclude those who do not align with their narrow vision. Even though the party is to be nationwide, it is clearly limited. For instance, the assumption that everyone involved in the celebration has access to a backyard where they could have tea and scones excluded lower class citizens and residents, who live primarily in apartment blocks, from participating in this invented national ritual. It similarly neglects the fact that the Muslim British population was celebrating Ramadan, which automatically excluded them from the daytime, food-based merriment.

(Original post by Pazzy (@pazzypants), “Anyone doing this on Friday then?” Twitter, May 5, 2020, 6:45 p.m., https://twitter.com/pazzypants1970/status/1257730359204229121.)

A more prosaic form of everyday exclusion as an extension of foreign policy is embodied through policing the conjugal order (George 2017; Peterson 1994; Yuval-Davis 1996). Far-right nationalists often construe women as biological reproducers of the nation, an especially crucial resource in unsettled times. Feminist scholars of nationalism have extensively discussed the connections between gender, intercourse, and national identity (Yuval-Davis, Anthias, and Campling 1989; McClintock 1993; Mayer 2012), where women need to produce new members of national collectives as well as reproduce the boundaries of national groups through restrictions on sexual or marital relations. The international relations literature emphasizes rape as a weapon of war (Kirby 2013; Card 1996; Maedl 2011), showing how sexual relations are often understood in terms of power and domination, equating the (often female) body of the population with the body of the state that needs to be forcibly invaded (Rohrer 1995). In other words, everyday foreign policy is gendered, ever-present, and affects women differently than men.

One of the more infamous everyday foreign policy practices related to the policing of conjugal order can be exemplified with wide-spread shaving of women’s hair in France after D-Day in 1944, another instance of unsettled times and practices that were aimed at reestablishing the status quo. The women who were publicly humiliated and often beaten up in the process were accused of *collaboration horizontale* with the invading German army (Virgili 1995). Unsurprisingly, *tondeurs*, the head shavers, often were not members of the Resistance but men who collaborated with the Nazi forces (François 2006). This scapegoating of women after D-Day is especially jarring given that other types of collaboration perpetrated by the male compatriots were hardly acknowledged or shamed in a similar way. For instance, the Vel d’Hiv deportation of Jews in Paris was carried and planned primarily by the French state—a fact fully acknowledged only in 2017 by a French president. As Beevor (2012) notes, exclusive revenge on women represented a form of recompense for the frustrations and sense of impotence among men humiliated by their country’s occupation. Thus, the more extreme practice of head shaving could be interpreted as an embodied amplification of xenophobia meant to reestablish an international structure where Germans (and everyone associated with them) were hierarchically inferior to the French and in this way signified the return to normalcy.

More recently, Russia’s announcement of sanctions against Turkey after the downing of a Russian fighter jet by the Turkish army on November 24, 2015, led Russian nationalists to recall the Russian-Turkish war of 1877–1878 (where the Russian Empire almost captured Istanbul) and its hero, General Skobelev, as reassurance that the country’s status and population would remain intact and superior to Turkey. Enemy embodiment continued with conjugal policing in the form of widespread harassment of Russian women who were married to Turkish citizens. This is yet another sign that everyday foreign policy requires autobiographical continuity to maintain ontological security for the population (Subotić 2016) with everyday foreign policy practices becoming crucial assertions of normality in unsettled times.

These various practices of gendering the nation by way of conjugal policing, enemy embodiment, and mobilization of collective memory in unsettled times converge in the impact of COVID-19. Women disproportionately bear the burden of the virus, amplified by foreign policies that undervalue international medical expertise. Women are more often at risk of infection, in part because they are more likely to be frontline healthcare workers. At the epicenter of the pandemic in Hubei, China, more than 90% of healthcare workers are women (Wenham, Smith, and Morgan 2020). The situation is not much different in the UK, where women are 77% of frontline healthcare workers (Norman 2020). The pandemic has disproportionately increased women’s exposure to domestic violence, burdens in the informal economy, risk of unemployment, and poverty (UN Women 2020)—consequences that were amplified by governments that failed to act to slow the outbreak. The effect of the pandemic thus interacts with, and reinforces, the gendered structure of labor markets as well as the gendered nature of state power. In the search for a visible enemy to explain the pandemic’s disruption of normal life, (mainly female) nurses and doctors have been targeted by disinformation and physical abuse. In Mexico, risk of infection is compounded by violence as nurses and doctors have been accused of spreading the virus and even attacked in the street (Nuño 2020). British NHS nurses and emergency workers suffered about 50 attacks per day, including being spat and coughed on, motivated by claims that they were spreading the virus. Similar attacks have been reported in the USA, India, the Philippines, Australia, and China (*The Economist* 2020).

### Comparing and Projecting Normality Online

As suggested by the discussion above, foreign policy extends into everyday life through war rhetoric, gendered practices, and articulations of historical or great power status. Even in unsettled times, people need to maintain their everyday foreign policy routines: tweeting with and about the nation, identifying enemies and allies, asserting their country’s status in the world, having long discussions on social media about which preposition to use with the country of Ukraine in Russian^10^, ordering a “rossiano” instead of an americano in Russia, or eating “freedom fries” instead of French fries in the USA. Given that (international) structures are in flux, everyday practices are meant to rediscover them. Like settled times when structures are fixed and practices are prompted by them, people need a sense of certainty that routines can offer. In times of social isolation, and with the inability to participate in conventional nation-building exercises such as parades and public commemoration, the need for ontological security that these rituals offer is greater, and projecting a state identity is an integral part of it. Particularly in the context of the pandemic, when many routines have been disrupted, it is not surprising that social networks serve as platforms for establishing the friend-foe dichotomy and performing familiar state identities.

When things are unsettled, other countries may provide examples of what constitutes normal. In online debates, people are usually split on whether their country needs to maintain its great power status by providing humanitarian aid to other countries. Similarly, many discussions have been devoted to whether China has replaced the USA as a global hegemon, given that it has provided shipments of medical equipment to Italy. On Russian social media, patriotic groups devote much attention to reports on how Russia is helping out Italy or the USA and how some countries cannot survive a pandemic without Russia’s help, thus reproducing the soft power narrative offered by the government. In this sense, everyday discursive practices are a creative engagement with the governmental rhetoric, sometimes challenging and sometimes amplifying a governmental stance to an extreme. For instance, the USA is frequently mocked, where users point out that the country’s behavior does not correspond to its movie image.

**Figure 5.**
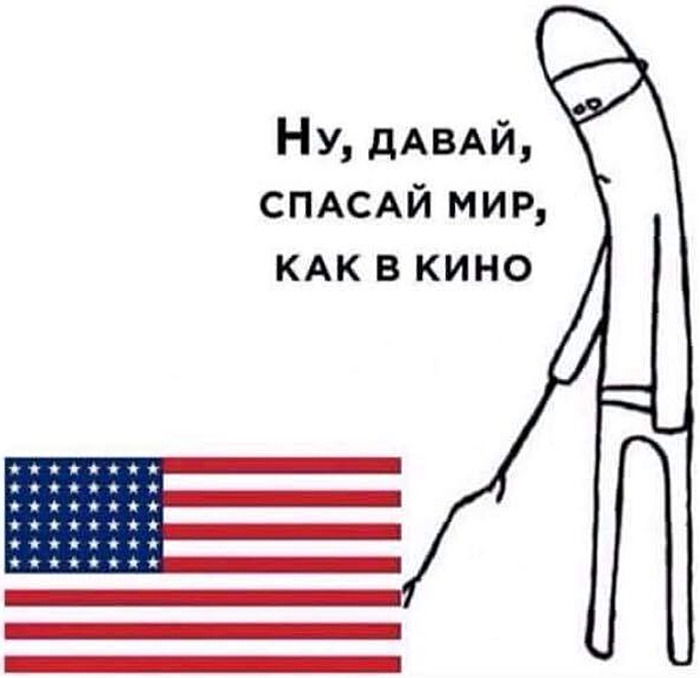
“Come on, save the world, like in the movies.”

One of the groups on Russian social media is vehemently anti-Ukrainian and pro-Crimea annexation. The meme from their group (figure 5) reflects the disconnect between the great power projection most people are used to around the world. At the same time, in the USA, social media users lament the fact that their country is unable to provide world leadership during the pandemic and has ceded its status as world hegemon to China (Tharoor 2020).

(Originally posted by Pavel Ptitsyn [@paulisru], Photo on VK, May 8, 2020, 10:06 p.m., https://m.vk.com/wall-41232698_5213011.)

## Conclusion: Everyday Nationalism and Unsettled Futures

The community of the nation provides a safe harbor for many in the turbulent storms kicked up by unsettled times. We have argued that in unsettled times, such as those surrounding the outbreak of COVID-19, ontological insecurity drives people to seek stability and comfort in the social practices that create solidarity out of routines and restoring the familiar. As we note, despite the virus’ impact as a truly global crisis, people often call for national solutions and cling hard to practices that sustain a sense of normalcy by invoking national bonds when all other sources of order appear to have broken down. Unsettled times may lead to calls for national unity and communal effort to overcome shared challenges and restore normal order. Calls for all citizens to wear protective gear, refrain from hoarding food, or make symbolic displays of support may be seen as extraordinary measures undertaken by all in order to safeguard and preserve a perceived national way of life. In other cases, people build solidarities through radical actions that seek to enforce normalcy by reinforcing hierarchies of dominance or excluding those labeled as outsiders, who violate their conceptions of what national normality ought to be.

In suggesting that the everyday nationalism of unsettled times seeks a return to a stable status quo, our conclusion departs from established theory in the field of social movements that suggests unsettled times produce mandates for broad change. Charles Tilly (1981) argues that times of crisis give rise to new forms of solidarity and association that transform systems. Such transformations arise when crisis broadens opportunities for mobilization around altering the status quo. While ordinary time provides micro-windows for systemic change, periods of crisis open macro-windows, enlarging opportunities for dramatic paradigm shifts. These studies suggest that the urgency and fear produced by unsettled times discredits those in charge and creates an environment “uniquely conducive to the passage of reform” (Keeler 1993, 441). Importantly, these studies theorize that disruptions to quotidian routines are especially effective in mobilizing collective action. In moments where the structure of social control and the status quo fracture, the prospect of loss leads ordinary people to seek more risk in attempts to enact change that minimize harm (Snow et al. 1998). Social movement theory suggests that the macro-windows for policy change opened by unsettled times present opportunities for political actors to exploit popular dissatisfaction with the status quo in order to enact their own political agendas (Kingdon 2003; Boin, ’t Hart, and McConnell 2009). Fringe political actors may seize these moments where crisis presents opportunity to push extreme ideas into mainstream discourse (Koopmans and Olzak 2004; Rovny 2013). Rather than seeking a return to normalcy, unsettled times motivate actors to upend the perceived normal and enact change.

We suggest that in the context of unsettled times, the desire to assert a sense of normality becomes *itself* a radical demand. As ordinary people attempt to find ontological security in their appeals to the community of the nation, ideas concerning what is normal and who is included in normality become normative assertions of the nation as these participants believe it *ought to be*, or *should have been* if not for the intervening crisis. In asserting such prescriptive notions of what the nation encompasses, those calling for normality assert a vision of radical stability.

Moreover, we contend that observations of unsettled times allow us to more fully understand how the nation is reproduced and how ordinary people determine what the nation is, and what it is not. Looking to unsettled times explores the everyday at what Jon Fox refers to as “the edges of the nation,” defined as those “places, times and contexts where the nation is on the periphery” (2017, 33). We propose that because the disruption of unsettled times heightens the salience of what may, under ordinary circumstances, have been tacit or taken for granted practices that reproduce or invoke the nation, they act as a large-scale breaching experiment. For instance, examinations of national practices enacted in the midst of crisis may provide opportunities to understand whether mass publics accept the official representations of the nation—often expressed in inclusive, or multicultural, terms—or offer alternative and more exclusory understandings of who is included in solidarity. Studying the way in which the nation is reproduced in times of crisis illuminates ordinary citizens’ perception of the function of the nation and the capacities possessed by the national community to address instability. In this sense we may hope to learn more about why, when faced with global problems, solutions frequently take national forms.

These conclusions suggest new ways to understand everyday nationalism as a transnational phenomenon. Much of the scholarship in the discipline rests on ethnographic examination of single cases. As our examination of the everyday response to COVID-19 has shown, in unsettled times ordinary people seek national solutions to global problems. We have also shown that the everyday practices of the nation undertaken by ordinary people also encompass understandings of foreign policy and international engagement. We propose that examinations of the everyday amid noisy or unsettled times provides opportunities to understand how nationalizing frames diffuse across borders even on an everyday level.

Looking beyond the context of COVID-19, we note that examinations of responses to the virus present lessons for how we might observe other, slower-moving challenges that likewise threaten notions of stability and daily practices of national significance. The growing threats posed by climate change present one example. Activists and scholars alike maintain that climate change presents universal challenges and requires solutions on a global scale (Conversi 2020). However, much like COVID-19, solutions to climate change largely involve implementation of policy on a national level. While a great deal of the collective action surrounding climate change aims to encourage action by national governments, activists also suggest that prevailing over climate change will require dramatic alterations to daily life. The lessons learned through observations of everyday nationalism during the response to COVID-19 provide a framework through which we might interpret responses to the climate crisis.

Though the foundational works in the field initially sought to understand how ordinary people interacted with the nation in times of low salience, where national identity fades into the background of ordinary life, the noisy moments of unsettled times still benefit from a close examination of the everyday. After all, even during great calamity, daily life not only persists but becomes a powerful call to action.

## Notes

1 Banal nationalism, most often associated with Michael Billig’s (1995) landmark work, focuses on the ways that the nation is reproduced in citizens’ daily lives mainly by way of the state and the media. Everyday nationalism (Fox and Miller-Idriss 2008; Goode and Stroup 2015) focuses on citizens’ agency not just in reproducing national repertoires and narratives but also in manipulating and challenging them. Increasingly, scholars have focused on the interaction between banal and everyday nationalism (Skey and Antonsich 2017; Goode 2020a).

2 Victor Petrov (@VictorPPetrov), “Academic news: Eric Hobsbawm’s new volume ‘The Age of Eternity: March 2020-March 2020’ is coming out soon,” Twitter, April 1, 2020, 2:23 a.m., https://twitter.com/VictorPPetrov/status/1245159893226717184.

3 See Ichijo (2020) for an exhaustive review of the literature on food and nationalism.

4 Depictions of panic buying in other nations via traditional and social media also reinforced a normal sense of international order by highlighting the distinctive items that national Others were hoarding, while eliding cases that appeared less characteristic. Hence, international media coverage of Americans’ hoarding of firearms neglected to mention a related surge in gun sales in Canada (Schwartz 2020).

5 See, for example, “World War Two Foods that Changed the Way We Eat,” BBC, n.d., https://www.bbc.co.uk/food/articles/world_war_two_foods.

6 A similar verb, *hamsteren*, was common in the Netherlands.

7 BBC Breakfast, “A message to people who are panic buying,” Video on Facebook, March 20, 2020, https://www.facebook.com/bbcbreakfast/videos/209680826924299/.

8 See Yablokov (2018) for an authoritative account of the role of conspiracy theories in contemporary Russia.

9 It is notable that nobody noticed or seemed concerned by the flyers’ lack of any attributable source, despite their being widely circulated through regular mail and on social media. Their content appears to have been coordinated by the VE Day 75 Pageantmaster (https://www.veday75.org/programme/), though the flyer does not appear on its website. In this fashion, the flyer seemed to emerge as a spontaneous, organic, and unquestioned expression of national belonging.

10 In the Russian language, one who uses the preposition *na* instead of *v* in relation to Ukraine is more likely to be perceived to harbor anti-Ukrainian views and support the annexation of Crimea.

## References

[r1] Acuto, Michele. 2014 “Everyday International Relations: Garbage, Grand Designs, and Mundane Matters.” International Political Sociology 8 (4): 345–362.

[r2] Adler, Emanuel, and Vincent Pouliot. 2011 “International Practices.” International Theory 3 (1): 1–36.

[r3] Adler-Nissen, Rebecca. 2016 “Towards a Practice Turn in EU Studies: The Everyday of European Integration.” Journal of Common Market Studies 54 (1): 87–103.

[r4] Adler-Nissen, Rebecca, and Vincent Pouliot. 2014 “Power in Practice: Negotiating the International Intervention in Libya.” European Journal of International Relations 20 (4): 889–911.

[r5] Balmforth, Tom. 2015 “On Display At ‘Made-in-Russia’ Fair: Hope, Patriotism, and Crimean Wine.” Radio Free Europe/Radio Liberty, September 16, 2015. https://www.rferl.org/a/russia-patriotic-trade-fair-import-substitution/27252333.html. (Accessed May 22, 2020.)

[r6] Balogun, Oluwakemi M. 2012 “Cultural and Cosmopolitan: Idealized Femininity and Embodied Nationalism in Nigerian Beauty Pageants.” Gender and Society 26 (3):357–381.

[r7] *BBC News*. 2020a. “UK Applauds NHS Staff and Other Key Workers.” April 2, 2020. https://www.bbc.com/news/uk-52140383. (Accessed May 22, 2020.)

[r8] *BBC News*. 2020b. “What’s behind the Anti-Lockdown Protests in US?,” April 21, 2020. https://www.bbc.com/news/world-us-canada-52359100. (Accessed May 22, 2020.)

[r9] Beevor, Antony. 2012 D-Day: The Battle for Normandy. UK: Penguin.

[r10] Benner, Tom. 2020 “Going Viral: Asia Takes on the Coronavirus with Songs, Dances.” Al Jazeera, March 11, 2020. https://www.aljazeera.com/news/2020/03/viral-asia-takes-coronavirus-songs-dances-200311063529703.html. (Accessed May 22, 2020.)

[r11] Berdahl, Daphne. 1999 Where the World Ended: Re-Unification and Identity in the German Borderland. Berkeley: University of California Press.

[r12] Bieber, Florian. 2020 “Global Nationalism in Times of the COVID Pandemic.” *Nationalities Papers.* Published online ahead of print April 27, 2020. doi:10.1017/nps.2020.35.

[r13] Billig, Michael. 1995 Banal Nationalism. London: Sage Publications.

[r14] Björkdahl, Annika, Martin Hall, and Ted Svensson. 2019 “Everyday International Relations: Editors’ Introduction.” Cooperation and Conflict 54 (2): 123–130.

[r15] Boin, Arjen, Paul ’t Hart, and Allan McConnell. 2009 “Crisis Exploitation: Political and Policy Impacts of Framing Contests.” Journal of European Public Policy 16 (1): 81–106.

[r16] Bonikowski, Bart. 2016 “Nationalism in Settled Times.” Annual Review of Sociology 42 (1): 427–449.

[r17] Braw, Elisabeth. 2020. “The Coronavirus Epidemic and Panic Buying: Follow the East German Example.” Royal United Services Institute, March 23, 2020. https://rusi.org/commentary/coronavirus-epidemic-and-panic-buying-follow-east-german-example. (Accessed May 22, 2020.)

[r18] Brophy, David. 2018 Review of *Silent Invasion: China’s Influence in Australia*, by Clive Hamilton. Australian Book Review, no. 400 (April). https://www.australianbookreview.com.au/abr-online/archive/2018/218-april-2018-no-400/4663-david-brophy-reviews-silent-invasion-china-s-influence-in-australia-by-clive-hamilton. (Accessed May 22, 2020.)

[r19] Brubaker, Rogers. 2004 Ethnicity Without Groups. Cambridge, MA: Harvard University Press.

[r20] Bueger, Christian, and Frank Gadinger. 2018 International Practice Theory. 2nd ed. New York, NY: Palgrave Macmillan.

[r21] Bulakh, Tetiana. 2018 “Made in Ukraine: Consumer Citizenship During EuroMaidan Transformations” In Identity and Nation Building in Everyday Post-Socialist Life, edited by Abel Polese, Jeremy Morris, Emilia Pawlusz, and Oleksandra Seliverstova, 73–90. London: Routledge.

[r22] Burgess, Adam, and Mitsutoshi Horii. 2012 “Risk, Ritual and Health Responsibilisation: Japan’s ‘Safety Blanket’ of Surgical Face Mask-Wearing: Surgical Face Mask-Wearing in Japan.” Sociology of Health and Illness 34 (8): 1184–1198. 22443378 10.1111/j.1467-9566.2012.01466.x

[r23] Caldwell, Melissa L. 2002 “The Taste of Nationalism: Food Politics in Postsocialist Moscow.” Ethnos: Journal of Anthropology 67 (3): 295–319.

[r24] Card, Claudia. 1996 “Rape as a Weapon of War.” Hypatia 11 (4): 5–18.

[r25] Cohen, Nick. 2020 “Coronavirus Panic Buying Is Turning Tories into Socialists.” The Spectator, March 20, 2020. https://www.spectator.co.uk/article/Coronavirus-panic-buying-is-turning-Tories-into-socialists. (Accessed May 22, 2020.)

[r26] Comaroff, John L., and Jean Comaroff. 2009 Ethnicity, Inc. Chicago, IL: University of Chicago Press.

[r27] Conversi, Daniele. 2020 “The Ultimate Challenge: Nationalism and Climate Change.” *Nationalities Papers.* Published online ahead of print March 23, 2020. doi:10.1017/nps.2020.18.

[r28] de Certeau, Michel. 1984 The Practice of Everyday Life. Berkeley: University of California Press.

[r29] DeSoucey, Michaela. 2010 “Gastronationalism: Food Traditions and Authenticity Politics in the European Union.” American Sociological Review 75 (3): 432–455.

[r30] Durkheim, Émile. 1915 The Elementary Forms of the Religious Life: A Study in Religious Sociology. Translated by Joseph Ward Swain. London: George Allen and Unwin Ltd.

[r31] Durkheim, Émile. 2008 “Anti-Semitism and Social Crisis (1899).” Sociological Theory 26 (4): 321–323.

[r32] Edensor, Tim. 2002 National Identity, Popular Culture and Everyday Life. Oxford: Berg Publishers.

[r33] Enloe, Cynthia. 1989 Bananas, Beaches and Bases. London: Pandora Press.

[r34] Eveleth, Rose. 2019 “Will Air-Filtering Face Masks Be the New Sunglasses?” Vox, March 19, 2019. https://www.vox.com/the-goods/2019/3/19/18262556/face-mask-air-filter-pollution-vogmask-airpop. (Accessed May 22, 2020.)

[r35] Feng, Shuo, Chen Shen, Nan Xia, Wei Song, Mengzhen Fan, and Benjamin J. Cowling. 2020 “Rational Use of Face Masks in the COVID-19 Pandemic.” The Lancet Respiratory Medicine 8 (5): 434–436. 32203710 10.1016/S2213-2600(20)30134-X PMC7118603

[r36] Ferré-Sadurní, Luis, and Jesse McKinley. 2020 “Alex Jones Is Told to Stop Selling Sham Anti-Coronavirus Toothpaste.” The New York Times, March 13, 2020. https://www.nytimes.com/2020/03/13/nyregion/alex-jones-coronavirus-cure.html. (Accessed May 22, 2020.)

[r37] Fox, Jon E. 2017 “The Edges of the Nation: A Research Agenda for Uncovering the Taken-for-Granted Foundations of Everyday Nationhood.” Nations and Nationalism 23 (1): 26–47.

[r38] Fox, Jon E., and Cynthia Miller-Idriss. 2008 “Everyday Nationhood.” Ethnicities 8 (4): 536–563.

[r39] Fox, Jon E., and Maarten Van Ginderachter. 2018 “Everyday Nationalism’s Evidence Problem.” Nations and Nationalism 24 (3): 546–552.

[r40] François, Dominique. 2006 Femmes tondues: la diabolisation de la femme en 1944: les bûchers de la Libération. Coudray-Macouard: Editions Cheminements.

[r41] Franklin, Marianne I. 2001 “Inside Out: Postcolonial Subjectivities and Everyday Life Online.” International Feminist Journal of Politics 3 (3): 387–422.

[r42] Gaufman, Elizaveta, and Dmytro Vovk. 2020 “Competition of Conspiracies: Conflicting Narratives of COVID-19 within the Grassroots Russian Orthodox Milieu.” Talk About: Law and Religion, March 30, 2020. https://talkabout.iclrs.org/2020/03/30/competition-of-conspiracies-conflicting-narratives-of-covid-19-within-the-grassroots-russian-orthodox-milieu/. (Accessed May 22, 2020.)

[r43] George, Nicole. 2017 “Policing ‘Conjugal Order’: Gender, Hybridity and Vernacular Security in Fiji." International Feminist Journal of Politics 19 (1): 55–70.

[r44] *Ghen Cô Vy*. 2020 Music Video. Min Official (YouTube Channel), February 23, 2020. https://www.youtube.com/watch?v=BtulL3oArQw. (Accessed May 22, 2020.)

[r45] Gohil, Necha Singh, and Dawinder S. Sidhu. 2007 “The Sikh Turban: Post-911 Challenges to This Article of Faith.” Rutgers Journal of Law and Religion 9 (2): 1–62.

[r46] Goode, J. Paul. 2016 “Love for the Motherland (or Why Cheese Is More Patriotic than Crimea).” Russian Politics 1 (4): 418–449.

[r47] Goode, J. Paul. 2020a. “Becoming Banal: Incentivizing and Monopolizing the Nation in Post-Soviet Russia.” *Ethnic and Racial Studies.* Published online ahead of print April 8, 2020. doi:10.1080/01419870.2020.1749687.

[r48] Goode, J. Paul. 2020b. Russia and Digital Surveillance in the Wake of COVID-19. PONARS Eurasia Policy Memo 650. Washington, D.C http://www.ponarseurasia.org/memo/russia-and-digital-surveillance-wake-covid-19. (Accessed May 22, 2020.)

[r49] Goode, J. Paul, and David R. Stroup. 2015 “Everyday Nationalism: Constructivism for the Masses.” Social Science Quarterly 96 (3): 717–739.

[r50] Gordenker, Alice. 2014 “Face Masks.” The Japan Times, November 21, 2014. https://www.japantimes.co.jp/news/2014/11/21/reference/face-masks/. (Accessed May 22, 2020.)

[r51] Gries, Peter Hays, Derek Steiger, and Tao Wang. 2016 “Popular Nationalism and China’s Japan Policy: The Diaoyu Islands Protests, 2012–2013.” Journal of Contemporary China 25 (98): 264–276.

[r52] Haddad, Yvonne Yazbeck. 2007 “The Post-9/11 Hijab as Icon.” Sociology of Religion 68 (3): 253–267.

[r53] Halikiopoulou, Daphne, Steven Mock, and Sofia Vasilopoulou. 2013 “The Civic Zeitgeist: Nationalism and Liberal Values in the European Radical Right.” Nations and Nationalism 19 (1): 107–127.

[r54] Hamilton, Clive. 2018 Silent Invasion: China’s Influence in Australia. Victoria, Australia: Hardie Grant Books.

[r55] Hansen, Lene. 2016 “Discourse Analysis, Post-Structuralism, and Foreign Policy” In Foreign Policy: Theories, Actors, Cases, edited by Steve Smith, Amelia Hadfield, and Tim Dunne. Oxford: Oxford University Press 10.1093/hepl/9780198708902.003.0005. (Accessed May 23, 2020.)

[r56] Hauerwas, Stanley. 2015 Performing the Faith: Bonhoeffer and the Practice of Nonviolence. Eugene, OR: Wipf and Stock Publishers.

[r57] Heller, Jeffrey. 2020 “Coronavirus Crisis Stoking Anti-Semitism Worldwide - Report.” Reuters, April 20, 2020. https://uk.reuters.com/article/uk-health-coronavirus-israel-antisemitis-idUKKBN222198. (Accessed May 22, 2020.)

[r58] Ho-him, Chan. 2020 “Two Arrested after Armed Gang Makes Run for Toilet Rolls in HK$1,600 Heist.” South China Morning Post, February 17, 2020. https://www.scmp.com/news/hong-kong/law-and-crime/article/3050907/armed-gang-steals-hk1000-toilet-paper-coronavirus. (Accessed May 22, 2020.)

[r59] Horvat, Srećko. 2020 “The Great Fear of Coronavirus: Panic! In the Supermarket.” Open Democracy, March 4, 2020. https://www.opendemocracy.net/en/can-europe-make-it/great-fear-coronavirus-panic-supermarket/.

[r60] Humphrey, Caroline. 2002 The Unmaking of Soviet Life: Everyday Economics after Socialism. Ithaca, NY: Cornell University Press.

[r61] Ichijo, Atsuko. 2020 “Food and Nationalism: Gastronationalism Revisited.” Nationalities Papers 48 (2): 215–223.

[r62] Jackson, Susan, and Christopher Laucht. 2020 “Soldiering a Pandemic: The Threat of Militarized Rhetoric in Addressing Covid-19.” History and Policy, April 24, 2020. http://www.historyandpolicy.org/opinion-articles/articles/soldiering-a-pandemic-the-threat-of-militarized-rhetoric-in-addressing-covid-19. (Accessed May 22, 2020.)

[r63] Jeung, Russell. 2020a. Incidents of Coronavirus Discrimination, March 19–25, 2020: A Report for A3PCON and CAA. 1. Stop AAPI Hate. Los Angeles, CA: Asian Pacific Policy and Planning Council, and Chinese for Affirmative Action.

[r64] Jeung, Russell. 2020b. Incidents of Coronavirus Discrimination, March 25–April 1, 2020: A Report for A3PCON and CAA. 2. Stop AAPI Hate. Los Angeles, CA: Asian Pacific Policy and Planning Council, and Chinese for Affirmative Action.

[r65] Jones, Rhys, and Peter Merriman. 2009 “Hot, Banal and Everyday Nationalism: Bilingual Road Signs in Wales.” Political Geography 28 (3): 164–173.

[r66] Kaye, Byron, and Chris Gallagher. 2020 “Rationing and Robbery: Coronavirus Outbreak Sparks Toilet Roll Panic.” Reuters, March 6, 2020. https://www.reuters.com/article/us-health-coronavirus-toiletpaper-idUSKBN20T0YG. (Accessed May 22, 2020.)

[r67] Keeler, John T. S. 1993 “Opening the Window for Reform: Mandates, Crises, and Extraordinary Policy-Making.” Comparative Political Studies 25 (4): 433–486.

[r68] Kim, Jo. 2020 “The Chinese People Step Up to Enforce China’s Nationalist Propaganda.” The Diplomat, May 5, 2020. https://thediplomat.com/2020/05/the-chinese-people-step-up-to-enforce-chinas-nationalist-propaganda/. (Accessed May 22, 2020.)

[r69] Kingdon, John W. 2003 Agendas, Alternatives, and Public Policies. 2nd ed. New York: Longman.

[r70] Kinnvall, Catarina. 2004 “Globalization and Religious Nationalism: Self, Identity, and the Search for Ontological Security.” Political Psychology 25 (5): 741–767.

[r71] Kirby, Paul. 2013 “How Is Rape a Weapon of War? Feminist International Relations, Modes of Critical Explanation and the Study of Wartime Sexual Violence.” European Journal of International Relations 19 (4): 797–821.

[r72] Kong, Lily, and Brenda S. A. Yeoh. 1997 “The Construction of National Identity through the Production of Ritual and Spectacle: An Analysis of National Day Parades in Singapore.” Political Geography 16 (3): 213–239.

[r73] Koopmans, Ruud, and Susan Olzak. 2004 “Discursive Opportunities and the Evolution of Right‐Wing Violence in Germany.” American Journal of Sociology 110 (1): 198–230.

[r74] Kovalev, Alexey. 2020 “The Kremlin’s Virus Blocker.” Meduza.Io, April 10, 2020. https://meduza.io/en/feature/2020/04/10/the-kremlin-s-virus-blocker. (Accessed May 22, 2020.)

[r75] Levada-Tsentr. 2020 “Pandemiia coronavirusa [The Coronavirus Pandemic].” Levada.ru, March 26, 2020. https://www.levada.ru/2020/03/26/pandemiya-koronavirusa/.

[r76] Lynteris, Christos. 2018 “Plague Masks: The Visual Emergence of Anti-Epidemic Personal Protection Equipment.” Medical Anthropology 37 (6): 442–457. 30427733 10.1080/01459740.2017.1423072

[r77] Maedl, Anna. 2011 “Rape as Weapon of War in the Eastern DRC-The Victims’ Perspective.” Human Rights Quarterly 33 (1): 128–147.

[r78] Maqbool, Aleem. 2020 “Why So Much US Resistance to the Lockdown?” BBC News, April 27, 2020. https://www.bbc.com/news/world-us-canada-52417610. (Accessed May 22, 2020.)

[r79] Marcus, Jonathan. 2020 “US-China Contagion: The Battle behind the Scenes.” BBC News, March 24, 2020. https://www.bbc.com/news/world-52008453. (Accessed May 22, 2020.)

[r80] Maxwell, Alexander. 2014 Patriots Against Fashion: Clothing and Nationalism in Europe’s Age of Revolutions. New York: Palgrave Macmillan.

[r81] Mayer, Tamar. 2012 “Gender Ironies of Nationalism: Setting the Stage” In Gender Ironies of Nationalism: Sexing the Nation, edited by Tamar Mayer, 15–38. London: Routledge.

[r82] McClintock, Anne. 1993 “Family feuds: Gender, nationalism and the family.” Feminist Review 44 (1): 61–80.

[r83] McPhee, Eliza. 2020 “Man Armed with Whip Goes on Racist Rant at Chinese Consulate.” Daily Mail, April 2, 2020. https://www.dailymail.co.uk/news/article-8178421/Man-armed-WHIP-goes-racist-tirade-outside-Chinese-Consulate.html. (Accessed May 22, 2020.)

[r84] Meade, Amanda. 2020 “Are ‘Busloads’ of Shoppers Really Stripping Australia’s Regional Supermarkets Bare?” Guardian, March 21, 2020. https://www.theguardian.com/world/2020/mar/21/are-busloads-of-shoppers-really-stripping-australias-regional-supermarkets-bare. (Accessed May 22, 2020.)

[r85] Militz, Elisabeth, and Carolin Schurr. 2016 “Affective Nationalism: Banalities of Belonging in Azerbaijan.” Political Geography 54: 54–63.

[r86] Mitzen, Jennifer. 2006 “Ontological Security in World Politics: State Identity and the Security Dilemma.” European Journal of International Relations 12 (3): 341–370.

[r87] Mitzen, Jennifer, and Kyle Larson. 2017 “Ontological Security and Foreign Policy.” *Oxford Research Encyclopedia of Politics.* Published online August 22, 2017 10.1093/acrefore/9780190228637.013.458. (Accessed May 22, 2020.)

[r88] Mohdin, Aamna. 2020 “Pots, Pans, Passion: Britons Clap Their Support for NHS Workers Again.” Guardian, April 2, 2020. https://www.theguardian.com/world/2020/apr/02/pots-pans-passion-britons-clap-their-support-for-nhs-workers-again. (Accessed May 22, 2020.)

[r89] Morozov, Viatcheslav. 2002 “Resisting Entropy, Discarding Human Rights: Romantic Realism and Securitization of Identity in Russia.” Cooperation and Conflict 37 (4): 409–429.

[r90] Neumann, Iver B. 2002 “Returning Practice to the Linguistic Turn: The Case of Diplomacy.” Millennium 31 (3): 627–651.

[r91] Nikitinskii, Leonid. 2020 “Novoe nevezhestvo [The New Ignorance].” Novaia Gazeta, May 9, 2020. https://www.novayagazeta.ru/articles/2020/05/09/85307-novoe-nevezhestvo. (Accessed May 22, 2020.)

[r92] Norman, Jenna. 2020 “Gender and Covid-19: The Immediate Impact the Crisis Is Having on Women.” *British Politics and Policy* (blog), *The London School of Economics and Political Science.* April 23, 2020. https://blogs.lse.ac.uk/politicsandpolicy/gender-and-covid19/. (Accessed May 22, 2020.)

[r93] Nuño, Analy. 2020 “‘What’s Wrong with You Mexico?’ Health Workers Attacked amid Covid-19 Fears.” Guardian, April 23, 2020. https://www.theguardian.com/world/2020/apr/23/mexico-health-workers-attacked-covid-19-fears. (Accessed May 22, 2020.)

[r94] Oppel, Jr., Richard A. 2020 “For Some Buyers with Virus Fears, the Priority Isn’t Toilet Paper. It’s Guns.” New York Times, March 16, 2020. https://www.nytimes.com/2020/03/16/us/coronavirus-gun-buyers.html. (Accessed May 22, 2020.)

[r95] Oprysko, Caitlin. 2020 “Trump Drafts Everyday Americans to Adopt His Battlefield Rhetoric.” Politico, May 9, 2020. https://www.politico.com/news/2020/05/09/donald-trump-coronavirus-wartime-rhetoric-245566. (Accessed May 22, 2020.)

[r96] Peterson, V. Spike. 1994 “Gendered Nationalism.” Peace Review 6 (1): 77–83.

[r97] Prizel, Ilya. 1998 National Identity and Foreign Policy: Nationalism and Leadership in Poland, Russia and Ukraine. Cambridge, UK: Cambridge University Press.

[r98] Reckwitz, Andreas. 2002 “Toward a Theory of Social Practices: A Development in Culturalist Theorizing.” European Journal of Social Theory 5 (2): 243–263.

[r99] Reeskens, Tim, and Matthew Wright. 2013 “Nationalism and the Cohesive Society: A Multilevel Analysis of the Interplay among Diversity, National Identity, and Social Capital across 27 European Societies.” Comparative Political Studies 46 (2): 153–181.

[r100] Ringmar, Erik. 2012 “Performing International Systems: Two East-Asian Alternatives to the Westphalian Order.” International Organization 66 (1): 1–25.

[r101] Rogers, Katie, Lara Jakes, and Ana Swanson. 2020 “Trump Defends Using ‘Chinese Virus’ Label, Ignoring Growing Criticism.” New York Times, March 18, 2020. https://www.nytimes.com/2020/03/18/us/politics/china-virus.html. (Accessed May 22, 2020.)

[r102] Rohrer, Tim. 1995 “The Metaphorical Logic of (Political) Rape: The New Wor(l)d Order.” Metaphor and Symbol 10 (2): 115–137.

[r103] Rose, Emily M. 2015 The Murder of William of Norwich: The Origins of the Blood Libel in Medieval Europe. Oxford: Oxford University Press.

[r104] Rose, Joel. 2020 “Frustration Mounts with Stay-at-Home Orders as Weeks Turn To Months.” National Public Radio, April 20, 2020. https://www.npr.org/sections/coronavirus-live-updates/2020/04/20/838596667/frustration-mounts-with-stay-at-home-orders-as-weeks-turn-to-months. (Accessed May 22, 2020.)

[r105] Rovny, Jan. 2013 “Where Do Radical Right Parties Stand? Position Blurring in Multidimensional Competition.” European Political Science Review 5 (1): 1–26.

[r106] Rumelili, Bahar. 2015 “Identity and Desecuritisation: The Pitfalls of Conflating Ontological and Physical Security.” Journal of International Relations and Development 18 (1): 52–74.

[r107] Schwartz, Noah S. 2020 “Why Canadians and Americans Are Buying Guns during the Coronavirus Pandemic.” The Conversation, April 8, 2020. http://theconversation.com/why-canadians-and-americans-are-buying-guns-during-the-coronavirus-pandemic-135409. (Accessed May 22, 2020.)

[r108] Siu, Judy Yuen-man. 2016 “Qualitative Study on the Shifting Sociocultural Meanings of the Facemask in Hong Kong since the Severe Acute Respiratory Syndrome (SARS) Outbreak: Implications for Infection Control in the Post-SARS Era.” International Journal for Equity in Health 15 (1): 73. 27145823 10.1186/s12939-016-0358-0 PMC4855818

[r109] Skey, Michael. 2009 “The National in Everyday Life: A Critical Engagement with Michael Billig’s Thesis of Banal Nationalism.” The Sociological Review 57 (2): 331–346.

[r110] Skey, Michael. 2010 “‘A Sense of Where You Belong in the World’: National Belonging, Ontological Security and the Status of the Ethnic Majority in England.” Nations and Nationalism 16 (4): 715–733.

[r111] Skey, Michael. 2011 National Belonging and Everyday Life: The Significance of Nationhood in an Uncertain World. New York: Palgrave Macmillan.

[r112] Skey, Michael, and Marco Antonsich, eds. 2017 Everyday Nationhood: Theorising Culture, Identity and Belonging after Banal Nationalism. Palgrave Macmillan.

[r113] Skitka, Linda J. 2005 “Patriotism or Nationalism? Understanding Post-September 11, 2001, Flag-Display Behavior.” Journal of Applied Social Psychology 35 (10): 1995–2011.

[r114] Snow, David, Daniel Cress, Liam Downey, and Andrew Jones. 1998 “Disrupting the Quotidian: Reconceptualizing the Relationship Between Breakdown and the Emergence of Collective Action.” Mobilization: An International Quarterly 3 (1): 1–22.

[r115] Subotić, Jelena. 2016 “Narrative, Ontological Security, and Foreign Policy Change.” Foreign Policy Analysis 12 (4): 610–627.

[r116] Syed, Qutub, Will Sopwith, Martyn Regan, and Mark A. Bellis. 2003 “Behind the Mask. Journey through an Epidemic: Some Observations of Contrasting Public Health Responses to SARS.” Journal of Epidemiology and Community Health 57 (11): 855–856. 14600109 10.1136/jech.57.11.855 PMC1732315

[r117] Tang, Catherine So-kum, and Chi-yan Wong. 2004 “Factors Influencing the Wearing of Facemasks to Prevent the Severe Acute Respiratory Syndrome among Adult Chinese in Hong Kong.” Preventive Medicine 39 (6): 1187–1193. 15539054 10.1016/j.ypmed.2004.04.032 PMC7133369

[r118] Tavernise, Sabrina, and Richard A. Oppel Jr. 2020 “Spit On, Yelled At, Attacked: Chinese-Americans Fear for Their Safety.” New York Times, March 23, 2020. https://www.nytimes.com/2020/03/23/us/chinese-coronavirus-racist-attacks.html. (Accessed May 22, 2020.)

[r119] Tharoor, Ishan. 2020 “The World Doesn’t Want to Pick Between the U.S. and China.” Washington Post, April 28, 2020. https://www.washingtonpost.com/world/2020/04/28/world-doesnt-want-pick-between-us-china/. (Accessed May 22, 2020.)

[r120] *The Economist*. 2020 “Health Workers Become Unexpected Targets during Covid-19.” May 11, 2020. https://www.economist.com/international/2020/05/11/health-workers-become-unexpected-targets-during-covid-19. (Accessed May 22, 2020.)

[r121] Tilly, Charles. 1981 “Useless Durkheim” In As Sociology Meets History, edited by Charles Tilly, 95–108. New York: Academic Press.

[r122] UN Women. 2020 *The Impact of COVID-19 on Women.* Policy Brief. April 9, 2020. https://www.unwomen.org/-/media/headquarters/attachments/sections/library/publications/2020/policy-brief-the-impact-of-covid-19-on-women-en.pdf?la=en&vs=1406. (Accessed May 22, 2020.)

[r123] Urrestarazu, Ursula Stark. 2015 “‘Vienna Calling’: Diplomacy and the Ordering of Intercommunal Relations at the Congress of Vienna.” The Hague Journal of Diplomacy 10 (3): 231–260.

[r124] Van Rythoven, Eric. 2020 “What’s Wrong with the War Metaphor.” Duck of Minerva, April 5, 2020. https://duckofminerva.com/2020/04/whats-wrong-with-the-war-metaphor.html. (Accessed May 22, 2020.)

[r125] Vigdor, Neil. 2020 “Tennessee Brothers Who Hoarded Hand Sanitizer Settle to Avoid Price-Gouging Fine.” New York Times, April 22, 2020. https://www.nytimes.com/2020/04/22/us/hand-sanitizer-matt-colvin-noah-coronavirus.html. (Accessed May 22, 2020.)

[r126] Virgili, Fabrice. 1995 “Les “tondues” à la Libération: le corps des femmes, enjeu d’une réaproppriation.” Clio. Femmes*, Genre, Histoire* 1.

[r127] Weale, Sally. 2020 “Chinese Students Flee UK after ‘Maskaphobia’ Triggered Racist Attacks.” Guardian, March 17, 2020. https://www.theguardian.com/education/2020/mar/17/chinese-students-flee-uk-after-maskaphobia-triggered-racist-attacks. (Accessed May 22, 2020.)

[r128] Wenham, Clare, Julia Smith, and Rosemary Morgan. 2020 “COVID-19: The Gendered Impacts of the Outbreak.” The Lancet 395 (10227): 846–848. 10.1016/S0140-6736(20)30526-2 PMC7124625 32151325

[r129] Wibben, Annick T. R. 2016 Researching War: Feminist Methods, Ethics and Politics. London: Routledge.

[r130] Wilson, Bee. 2020 “Off Our Trolleys: What Stockpiling in the Coronavirus Crisis Reveals about Us.” Guardian, April 3, 2020. https://www.theguardian.com/news/2020/apr/03/off-our-trolleys-what-stockpiling-in-the-coronavirus-crisis-reveals-about-us. (Accessed May 22, 2020.)

[r131] Wodak, Ruth, and Michał Krzyżanowski. 2017 “Right-Wing Populism in Europe & USA: Contesting Politics & Discourse Beyond ‘Orbanism’ and ‘Trumpism.’ ” *Journal of Language and Politics* 16 (4): 471–484.

[r132] Yablokov, Ilya. 2018 Fortress Russia: Conspiracy Theories in the Post-Soviet World. Cambridge, UK: Polity Press.

[r133] Yuval-Davis, Nira. 1996 “Women and the Biological Reproduction of ‘the Nation.’ ” Women’s Studies International Forum 19 (1/2): 17–24.

[r134] Yuval-Davis, Nira, and Floya Anthias, eds. 1989 Woman, Nation, State. New York: Palgrave Macmillan.

[r135] Zelenaia, Dar’ia. 2020 “Grechka ischezaet v polden’ [Buckwheat Is Gone by Noon].” Novaia Gazeta, March 17, 2020. https://www.novayagazeta.ru/articles/2020/03/17/84350-grechka-ischezaet-v-polden. (Accessed May 22, 2020.)

